# Immunometabolic control of macrophage plasticity in wound healing: mechanistic insights and therapeutic opportunities

**DOI:** 10.3389/fimmu.2026.1793796

**Published:** 2026-07-13

**Authors:** Lijing Shi, Hongyou Zhou, Rui Zheng

**Affiliations:** 1 Department of Obstetrics and Gynecology, Lishui Central Hospital, Lishui, Zhejiang, China; 2 Department of Plastic Surgery, Lishui Central Hospital, Lishui, Zhejiang, China

**Keywords:** chronic tissue injury, inflammation, macrophage, metabolic reprogramming, tissue repair

## Abstract

Macrophages play a pivotal regulatory role in inflammation, tissue repair, and fibrosis through their dynamic changes in phenotype and function. The tissue microenvironment following injury induces alterations in key metabolic enzymes, signaling pathways, and metabolites within macrophages, thereby driving shifts in their phenotype and function. Early in acute injury, macrophages primarily rely on glycolysis and the pentose phosphate pathway, transitioning to a pro-inflammatory phenotype. Persistent activation of pro-inflammatory macrophages can lead to tissue damage. As the metabolic microenvironment evolves, the expression of glycolysis-related genes is suppressed, while the expression of genes related to oxidative phosphorylation and the tricarboxylic acid cycle is upregulated, promoting the gradual shift of macrophages toward an anti-inflammatory phenotype. This process plays a crucial role in tissue repair and remodeling. However, sustained activation of anti-inflammatory macrophages may contribute to the development of fibrosis. Therefore, metabolic reprogramming of macrophages presents a novel potential therapeutic target for intervening in inflammatory injury and stromal fibrosis. The high plasticity of macrophages is essential for tissue repair and regeneration, as they regulate inflammation, promote angiogenesis, and facilitate extracellular matrix remodeling, thereby restoring tissue homeostasis. This capability holds promise for the treatment of various conditions, including chronic wounds, fibrotic diseases, and inflammatory disorders.

## 1 Introduction

Tissue repair and regeneration are crucial biological processes. When tissue is damaged by infection, toxicity, or mechanical injury, cells in the wound activate an inflammatory response by sensing damage-associated molecular patterns (DAMPs) and pathogen-associated molecular patterns (PAMPs) released by dying and dead cells, as well as invading organisms (1, 2). These pattern recognition molecules trigger a complex inflammatory response, including the recruitment, proliferation, and activation of various inflammatory cells, which collectively coordinate the repair process. Wound healing proceeds through four overlapping phases: hemostasis, inflammation, proliferation/repair, and remodeling (3). When inflammation is appropriately controlled, it resolves rapidly and tissue architecture is restored. However, when wound healing becomes chronic or dysregulated, two distinct pathological trajectories emerge. Defective repair manifests as chronic non-healing wounds. Excessive activation of tissue remodeling programs drives pathological fibrosis or scar formation. This process impairs normal tissue function and ultimately compromises physiological function (4, 5). Although various cell types are involved in tissue repair, macrophages, with their highly plastic programming abilities, play a central regulatory role at all stages, from the initial inflammatory response to inflammation resolution and tissue remodeling (6, 7). Therefore, macrophages represent a potential key therapeutic target for normal tissue repair.

Over the past few decades, research has increasingly focused on uncovering the molecular mechanisms that regulate macrophage plasticity and their roles in tissue repair and regeneration. After tissue injury, monocytes are recruited from the bone marrow to the injured area via chemokine gradients and adhesion molecules, and the number of these recruited cells typically exceeds the number of resident macrophages in the local tissue (8, 9). These recruited and resident macrophages not only undergo changes in number but also experience significant phenotypic and functional shifts in response to growth factors and cytokines in the local microenvironment (10, 11). Over the past decade, a clear consensus has emerged in macrophage biology. The field has moved away from the M1/M2 dichotomy that long dominated the literature. The expert consensus on macrophage activation nomenclature proposed by Murray et al. in Immunity in 2014 recommends a more rigorous approach. Macrophages should be described according to their inducing signals, expression markers, secreted cytokines, and direct effector functions (12). Accordingly, this article uses the three-type immunity framework, which is based on evolutionarily defined classes of threat, to discuss macrophage effector programs. These include type 1 immunity driven by IFN-γ and IL-12, type 2 immunity driven by IL-4 and IL-13, and type 3 immunity driven by IL-17. Importantly, all three immune types represent distinct inflammatory programs. They differ in the threats they target and the effector functions they execute, rather than reflecting a simple opposition between “pro-inflammatory” and “anti-inflammatory” states.

**Figure 1:**
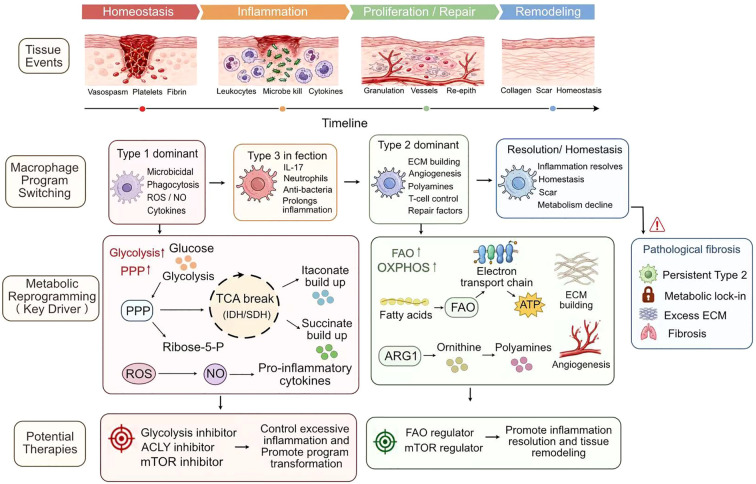
Metabolic reprogramming of macrophages orchestrates inflammation resolution, tissue repair, and fibrotic outcomes during wound healing. Wound healing proceeds through overlapping phases of hemostasis, inflammation, proliferation and repair, and remodeling. During this process, macrophages coordinate immune responses, angiogenesis, extracellular matrix remodeling, and tissue homeostasis through phenotypic switching and metabolic reprogramming. In early injury, macrophages predominantly adopt a pro-inflammatory Type 1-like program. This state is marked by enhanced glycolysis and pentose phosphate pathway activity, disrupted TCA cycle flux, and accumulation of metabolites such as succinate and itaconate. These changes promote ROS, NO, and pro-inflammatory cytokine production, thereby supporting pathogen clearance, phagocytosis, and inflammatory amplification. A subset of macrophages may also display a Type 3-like inflammatory regulatory program, which promotes IL-17 signaling, neutrophil recruitment, and antimicrobial defense. As healing progresses, macrophages shift toward a Type 2-like reparative program. This transition is associated with increased fatty acid oxidation and oxidative phosphorylation, which support ATP production, ARG1–ornithine–polyamine metabolism, angiogenesis, extracellular matrix deposition, granulation tissue formation, and re-epithelialization. Persistent Type 2-like activation may drive metabolic lock-in, excessive extracellular matrix deposition, and pathological fibrosis. Targeting stage-specific metabolic nodes, including glycolysis, ACLY, mTOR, fatty acid oxidation, and oxidative phosphorylation, may help control inflammation, promote resolution, and improve tissue remodeling. Abbreviations: PPP, pentose phosphate pathway; TCA, tricarboxylic acid cycle; ROS, reactive oxygen species; NO, nitric oxide; FAO, fatty acid oxidation; OXPHOS, oxidative phosphorylation; ECM, extracellular matrix; ARG1, arginase 1; ACLY, ATP-citrate lyase; mTOR, mechanistic target of rapamycin.

Yan et al. focused on signaling pathways and the role of macrophages in tissue repair and regeneration (13). However, their Review did not systematically explain how metabolic reprogramming drives the dynamic transition of macrophage effector programs. Nor did it examine how this transition determines repair outcomes. This Review addresses this gap from the perspective of metabolic reprogramming. We summarize the evidence showing how glycolysis, oxidative phosphorylation and the tricarboxylic acid cycle regulate the transition from type 1 microbicidal effector programs to type 2 tissue-remodeling effector programs. Each macrophage effector function, including phagocytosis, migration, cytokine secretion and tissue remodeling, is supported by a specific and targetable metabolic program. Defining the direct mechanistic links between metabolic reprogramming and effector output will deepen our understanding of macrophage biology. It will also provide a rationale for metabolism-targeted therapeutic strategies. This Review is organized into three sections. The first section outlines macrophage plasticity and effector-program diversity. It introduces the type 1, type 2 and type 3 immunity framework based on evolutionarily defined threat classes. It then discusses the dynamic roles of macrophages across the four stages of tissue repair and examines how functional dysregulation contributes to disease through defective repair or pathological fibrosis. The second section focuses on the signaling pathways that regulate macrophage effector programs. It emphasizes how these pathways control metabolic reprogramming and shape macrophage functional states, with a dedicated discussion of mTOR as a metabolic hub. The third section examines the therapeutic potential of targeting macrophage metabolic reprogramming and effector programs. It reviews current strategies for modulating the wound and injury microenvironment and considers future directions, with a focus on macrophage functional control in chronic wounds, fibrotic diseases and inflammatory disorders (**Figure 1**).

## 2 Macrophage effector program diversity and metabolic reprogramming

Macrophages are central components of the innate immune system. They show marked plasticity, which enables them to respond to changes in the tissue microenvironment and execute diverse functions. This section focuses on recent advances in macrophage effector-program diversity, with particular emphasis on the mechanisms of metabolic reprogramming. It further discusses the functional relevance of these processes in health and disease. Particular attention is given to tissue-specific adaptation and its role in trained immunity.

### 2.1 Macrophage effector programs: from historical classification to a threat-defined functional framework

For many years, macrophage biology used a binary classification system based on *in vitro* stimulation conditions to describe macrophage activation states. In this historical framework, macrophages activated by IFN-γ and lipopolysaccharide (LPS) were defined as historically termed “classically activated” macrophages. Macrophages induced by IL-4 and IL-13 were defined as historically termed “alternatively activated” macrophages. This classification provided an early working model for macrophage biology. However, over the past decade, rapid advances in single-cell omics and accumulating evidence from *in vivo* genetic fate-mapping studies have led to a clear consensus. This simple dichotomy cannot capture the diversity and complexity of macrophage activation states *in vivo*. The expert consensus on macrophage activation nomenclature stated that macrophage activation exists along a continuum. Macrophage phenotypes change dynamically according to the specific combinations of signals provided by the microenvironment (12). Accordingly, macrophage activation states should be described functionally according to their inducing signals, expression markers, secreted effector molecules and direct effector functions. On this basis, macrophage effector programs can be organized into three categories according to their evolutionary purpose, namely the type of threat they are designed to control.

First, type 1 immunity-associated macrophages are driven by IFN-γ and IL-12. They respond to small intracellular pathogens, such as viruses and intracellular bacteria. Their core effector functions include reactive oxygen species (ROS) production, phagocytosis, lysosomal acidification through V-ATPase and cell migration (14, 15). These functions share a common demand for rapid ATP supply and NADPH regeneration. Thus, this program is supported by high glycolytic flux and pentose phosphate pathway (PPP) activity. At the same time, the macrophage tricarboxylic acid (TCA) cycle shows characteristic breaks at isocitrate dehydrogenase (IDH) and succinate dehydrogenase (SDH). These breaks lead to the accumulation of itaconate and succinate. Both metabolites contribute to antimicrobial defence and the regulation of inflammatory signaling (16, 17).

Second, type 2 immunity-associated macrophages are driven by IL-4 and IL-13. They respond to large extracellular parasites and allergens. Their core effector functions include extracellular matrix (ECM) construction and tissue remodeling, local macrophage expansion, T cell regulation, and the encapsulation and containment of threats, such as granuloma formation (18, 19). These functions are supported by fatty acid oxidation (FAO), oxidative phosphorylation (OXPHOS), and the arginase 1 (ARG1)-ornithine–putrescine, spermine and spermidine synthesis axis. It is important to state clearly that type 2 immunity is itself a distinct inflammatory program (20, 21). Its capacity to promote tissue repair in specific contexts reflects its effector-molecule profile, including ECM construction and polyamine-mediated support of proliferation. It does not mean that this program is inherently anti-inflammatory. Indeed, type 2 immunity-associated macrophages can drive type 2 inflammation and represent a central pathogenic force in tissue injury in atopic dermatitis and severe asthma (22).

Third, type 3 immunity-associated macrophages are driven by IL-17. They respond to extracellular bacteria and fungi. Their core effector functions include neutrophil chemotaxis, mediated by the induction of G-CSF, CXCL8/IL-8 and other chemokines, and the production of antimicrobial peptides (23). Under steady-state conditions, IL-17 also helps maintain mucosal barrier integrity. It does so by inducing tight-junction proteins and antimicrobial peptides (24). However, this barrier-protective function should not be interpreted as anti-inflammatory. IL-17 is intrinsically a pro-inflammatory cytokine that drives neutrophil-enriched inflammation. It is also a well-established pathogenic mediator in psoriasis and inflammatory bowel disease (25, 26). The metabolic support mechanisms of type 3 immunity remain under active investigation. Current evidence suggests that IL-17 signaling can enhance glycolysis and promote glutaminolysis, thereby supporting the efficient translation of neutrophil-recruiting factors (27, 28).

These three types of immunity represent distinct forms of inflammatory programming. They differ in the threats they target, the signaling environments that induce them, and the effector functions they execute. They should not be reduced to a simple opposition between “pro-inflammatory” and “anti-inflammatory” states. Macrophages dynamically tune their effector programs in response to local microenvironmental cues, including DAMPs, PAMPs, cytokines, metabolites and oxygen tension. They move along a continuous functional spectrum rather than switching between two discrete states. This dynamic balance is essential for coordinating an appropriate immune response. It also enables an ordered transition from inflammation to tissue repair and helps maintain tissue homeostasis. Disruption of this balance can lead to chronic inflammation, autoimmune disease, chronic non-healing wounds and organ fibrosis. Thus, understanding macrophage effector programs through a functional framework has become central to defining their regulatory mechanisms and interactions. It also provides a key strategy for understanding and treating immune-mediated diseases.

### 2.2 Macrophage activation states and their specific signatures

The development of single-cell technologies has profoundly revealed the continuous spectrum of macrophage activation states, vividly demonstrating their high adaptability to environmental signals and their multiple roles in both health and disease (29, 30). These findings confirm the adaptability of macrophages in different microenvironments, suggesting their diverse functional roles in both physiological and pathological processes.

Recent breakthroughs in macrophage research have significantly advanced our understanding, particularly regarding the profound impact of the tissue microenvironment on their development, phenotype shaping, and functional regulation (31, 32). Macrophages are ubiquitously present in all tissues, where they perform specialized functions tailored to the specific needs of their microenvironment. The latest studies show that tissue-specific factors, including metabolites and cell-to-cell interactions, play a critical role in shaping the transcriptional and epigenetic marks of resident macrophages, thereby driving the formation of subpopulations with specific functions (33, 34). For instance, the regulation of tissue-specific transcription factors shapes the enhancer landscapes of macrophages in different tissues, highlighting their specialized functional characteristics. The gut microbiome has also been confirmed as one of the core factors regulating the function of intestinal macrophages.

Several studies have shown that microbial metabolites such as short-chain fatty acids and taurine can regulate the phenotype and activity of intestinal macrophages, thereby promoting homeostasis and enhancing resistance to intestinal infections (30, 35). These findings highlight the importance of studying macrophages in their native tissue environment and reveal the limitations of using *in vitro* models to infer physiological states *in vivo* (36, 37). The tissue-specific imprinting of macrophages is not only critical for understanding immune regulatory mechanisms but also provides key insights into the pathogenesis of related diseases. Dysregulation of these tissue-specific regulatory mechanisms can lead to inflammation and metabolic imbalances.

### 2.3 Macrophage metabolic reprogramming and short-term plasticity

Short-term plasticity refers to the dynamic regulatory mechanism by which macrophages respond to changes in their microenvironment. The core driving forces behind this process include the rapid activation of signaling pathways, transcriptional and epigenetic reprogramming, as well as metabolic reprogramming (38, 39). When macrophages sense DAMPs, PAMPs, cytokines, chemokines, metabolites, and hypoxic signals through pattern recognition receptors such as Toll-like receptors (TLRs), NLRs, and cytokine receptors, they can quickly adjust their phenotype, metabolic state, and function within hours to days (40, 41). This process involves the activation and regulation of signaling pathways. It drives the dynamic transition between type 1 antimicrobial effector programs and type 2 tissue-remodeling effector programs (42). Macrophages can rapidly adjust their metabolism and function. This capacity enables them to exert antimicrobial and pro-inflammatory effects during the early inflammatory phase. It also allows them to initiate tissue regeneration during the repair phase and subsequently promote tissue remodeling and functional recovery. Together, these responses establish a dynamic balance that supports tissue repair (43, 44). In the type 1 immune effector state, macrophages mainly depend on glycolysis. They show a Warburg-like metabolic profile, which supports rapid ATP production despite low energetic efficiency. This state is also marked by disruption of the tricarboxylic acid cycle, succinate accumulation, stabilization of hypoxia-inducible factor-1α, and increased expression of type 1 effector molecules.

The glycolytic enzymes PFKFB3 and PKM2 can localize around the phagosomal membrane. There, they provide local ATP and NADPH to support phagosome maturation and NOX2-dependent ROS production. This spatial proximity directly couples metabolic flux to inflammatory function (45). By contrast, macrophages that shift towards a type 2 tissue-remodeling program mainly rely on oxidative phosphorylation. They use fatty acids and glutamine as major substrates, while maintaining an intact TCA cycle. Arginine is converted by arginase 1 into ornithine. Ornithine is then decarboxylated by ornithine decarboxylase 1 to generate putrescine, which further gives rise to spermine and spermidine. These polyamines are essential building blocks for cell proliferation. They also support fibroblast collagen synthesis, thereby linking macrophage metabolism directly to extracellular matrix construction (46, 47). At the same time, mitochondrial citrate is exported by SLC25A1 and cleaved by ATP-citrate lyase (ACLY) to generate acetyl-CoA. Acetyl-CoA feeds into two major downstream pathways. *De novo* fatty acid synthesis, mediated by acetyl-CoA carboxylase (ACC) and fatty acid synthase (FASN), supports membrane expansion. Histone acetylation, such as H3K27ac at pro-inflammatory gene loci, links TCA-cycle remodeling to epigenetic reprogramming (48, 49). In addition, immunometabolites such as itaconate not only exert antimicrobial effects but also act as signaling molecules that regulate oxidative stress and inflammation (50). .

**Figure 2:**
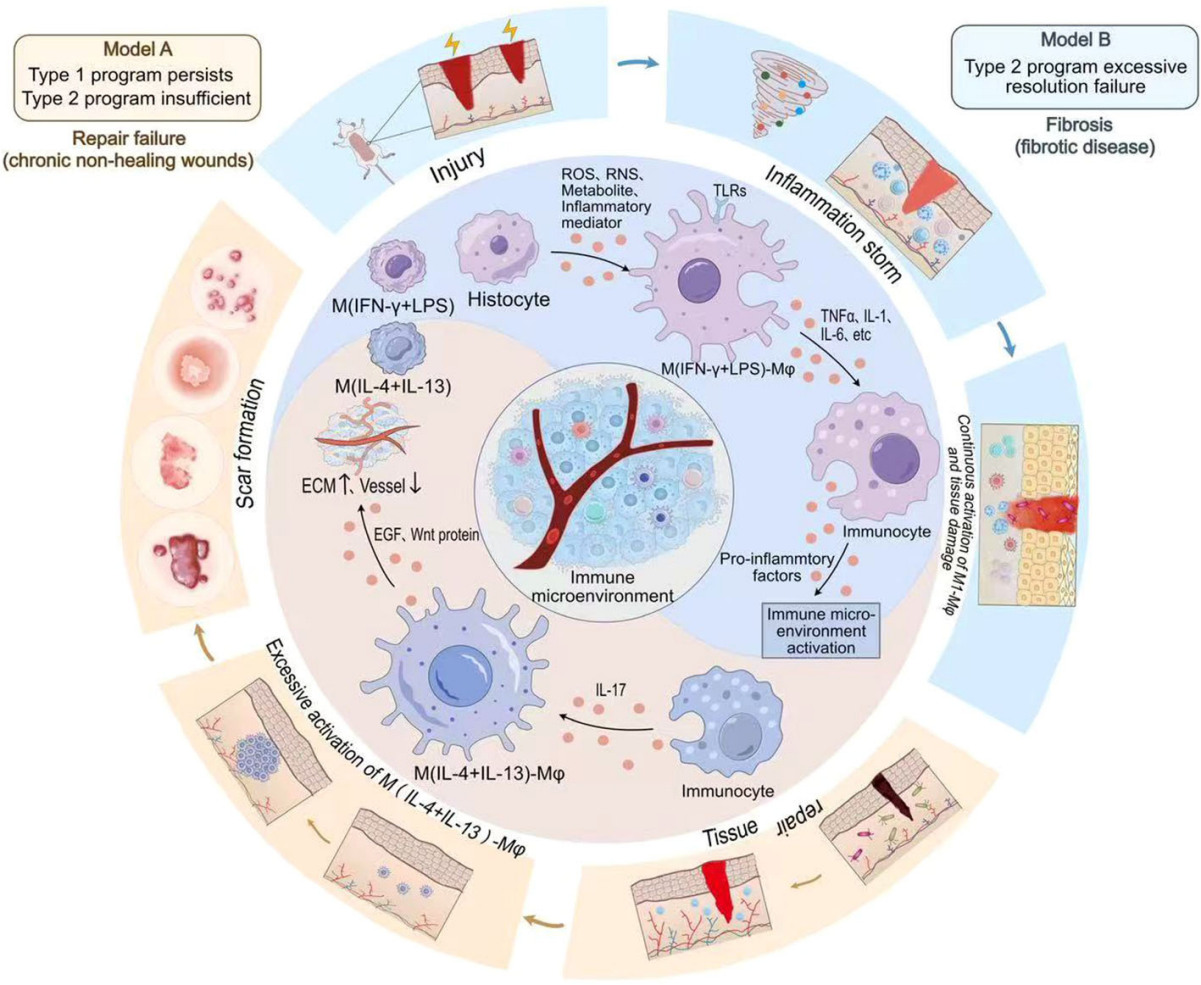
Macrophages play a key regulatory role in inflammation, tissue repair, and fibrosis. Following tissue injury, alterations in the immune microenvironment reshape the metabolic landscape of macrophages,reprogramming key metabolic enzymes, signaling pathways, and metabolite profiles. These changes drive macrophages toward the type 1 microbicidal effector program, characterized by elevated glycolytic flux and pro-inflammatory cytokine secretion. The altered metabolic milieu sustains this type 1 program in a hyperactivated state, fueling an inflammatory cascade that exacerbates tissue damage. During the subsequent repair phase, anti-inflammatory signals secreted by monocytes and other immune cells promote a gradual transition of macrophages toward the type 2 tissue remodeling effector program, which plays a pivotal role in resolving inflammation and orchestrating matrix reconstruction. However, persistent or dysregulated activation of this type 2 program-driven by sustained IL-4/IL-13 signaling and supported by fatty acid oxidation and polyamine synthesis can lead to excessive ECM deposition, pathological fibrosis, and scar formation. ROS, reactive oxygen species; RNS, reactive nitrogen species; M1 Mφ, M1 macrophages; M2 Mφ, M2 macrophages; TLRs, Toll-like receptors; TNFα, tumor necrosis factor-α; IL-17, interleukin-17; ECF, environmental cues; ECM, extracellular matrix.

Thus, a deeper understanding of macrophage short-term plasticity may have substantial clinical value. This is particularly relevant for therapeutic strategies that use drugs, biomaterials, or cell-based interventions to regulate effector-program switching (51). Modulating transient macrophage responses may promote tissue regeneration in organs such as the skin, nervous system, and myocardium. It may also reduce fibrosis in the liver, lung, and kidney, and improve chronic wound healing and inflammatory diseases such as arthritis and atherosclerosis (52, 53). Dysregulated macrophage effector programs show two distinct pathological patterns. The first is a failure to transition from a type 1 antimicrobial program to a type 2 tissue-remodeling program. This failure leads to defective repair. In chronic non-healing wounds, persistent glycolysis and pro-inflammatory activity directly impair tissue restoration. The second pattern is excessive activation or failed resolution of the type 2 tissue-remodeling program. This state drives excessive extracellular matrix deposition and collagen crosslinking. It is commonly observed in liver fibrosis, idiopathic pulmonary fibrosis, and cutaneous keloids (**Figure 2**). These two patterns occupy opposite ends of the macrophage effector-program imbalance spectrum. Together, they define a core pathogenic mechanism of repair-associated diseases.

## 3 Regulatory network of macrophage metabolic reprogramming

Recent studies have shown that the regulation of macrophage effector programs is a highly dynamic and complex biological process. It requires the integration of multiple signaling pathways (54). Macrophages sense signals from their microenvironment through receptors, which activate downstream signaling pathways, leading to the activation of transcription factors and epigenetic remodeling. This process drives metabolic pathway shifts, and metabolic changes further feedback-regulate signaling pathways and epigenetic mechanisms, ultimately promoting the expression of specific functional proteins (such as cytokines, enzymes, and growth factors) and facilitating rapid changes in phenotype and function (55, 56). This process is triggered by the activation of pattern recognition receptors (PRRs), cytokine receptors, and other surface molecules, which activate transcription factors and initiate metabolic reprogramming, thereby shaping the macrophage transcriptional profile (57, 58). Understanding the molecular mechanisms that regulate macrophage effector programs is essential for developing targeted therapeutic strategies that modulate macrophage function in inflammatory disease and tissue injury repair. This section discusses the key signaling pathways involved in the regulation of macrophage effector programs. It focuses on recent advances in metabolic reprogramming and their potential therapeutic implications.

### 3.1 mTOR as a central integrator of macrophage metabolic reprogramming

The mechanistic target of rapamycin (mTOR) is a highly conserved serine/threonine protein kinase. It acts as a central integrator of cellular metabolism and has a core role in macrophage metabolic reprogramming and effector-program control. mTOR exists in two structurally and functionally distinct multiprotein complexes: mTOR complex 1 (mTORC1) and mTOR complex 2 (mTORC2). mTORC1 is defined by subunits including RAPTOR, mLST8 and DEPTOR. It mainly controls anabolism, protein translation and autophagy inhibition. mTORC2 is defined by RICTOR, mSIN1 and PROTOR. It mainly regulates cytoskeletal remodeling, cell migration and cell survival (59). These two complexes have distinct but complementary regulatory roles in macrophage biology.

#### 3.1.1 mTORC1 as a central node for metabolic signal integration

mTORC1 activity is tightly controlled by upstream inputs. The first major input is growth factor signaling through the PI3K–AKT–TSC axis. Growth factors and cytokines, including insulin, colony-stimulating factor 1 (CSF-1) and IL-4, activate the PI3K–AKT pathway. AKT then phosphorylates and inhibits the TSC complex, composed of TSC1 and TSC2. This relieves TSC-mediated inhibition of the Rheb GTPase and thereby activates mTORC1. In macrophages associated with the IFN-γ/LPS-driven type 1 effector program, sustained AKT activation is required to maintain mTORC1 activity and downstream metabolic reprogramming (60).

The second major input is cellular energy sensing through the AMPK-TSC axis. AMP-activated protein kinase (AMPK) acts as a cellular energy sensor. It is activated when ATP is limited and the AMP/ATP ratio increases. AMPK inhibits mTORC1 through two mechanisms. It directly phosphorylates RAPTOR and suppresses mTORC1 activity. It also phosphorylates and activates the TSC complex, which indirectly inhibits mTORC1 through Rheb. This regulatory mechanism is especially important for the type 2 tissue-remodeling effector program. This program depends on fatty acid oxidation and oxidative phosphorylation to generate ATP efficiently. It therefore maintains a relatively low AMP/ATP ratio. This state partially releases mTORC1 inhibition and supports the moderate anabolic demand required for tissue remodeling.

#### 3.1.2 Role of mTORC1-driven metabolic reprogramming in the type 1 effector program

In macrophages associated with the IFN-γ/LPS-driven type 1 effector program, mTORC1 drives metabolic reprogramming through at least three parallel downstream pathways. The first pathway is cap-dependent translation of HIF-1α. mTORC1 phosphorylates eukaryotic translation initiation factor 4E-binding proteins, including 4E-BP1, 4E-BP2 and 4E-BP3. This releases eIF4E from 4E-BP-mediated inhibition and promotes cap-dependent translation of HIF-1α mRNA. This translational mechanism allows macrophages to rapidly accumulate HIF-1α protein even under oxygen-replete conditions, despite prolyl hydroxylase (PHD)-dependent degradation of HIF-1α. HIF-1α then activates the transcription of glycolytic genes, including Hk2, Pfkfb3 and Ldha, glucose transporter genes such as Slc2a1/GLUT1, and pro-inflammatory cytokine genes such as Il1b and Tnf (61). This axis provides a key molecular bridge linking nutrient signaling to the metabolic features of the type 1 effector program.

The second pathway is SREBP1/2-dependent *de novo* lipid synthesis. mTORC1 promotes the nuclear translocation and proteolytic activation of sterol regulatory element-binding proteins 1 and 2 (SREBP1/2) through an S6K-dependent mechanism. This induces the transcription of genes involved in *de novo* lipid synthesis, including FASN, ACC and ACLY (62). This pathway supplies the phospholipids and cholesterol required for phagosomal membrane formation and plasma membrane expansion. It also supports receptor signaling, including TLR4 signaling, by providing lipid raft components (63).

The third pathway is S6K-dependent ribosome biogenesis and protein translation. mTORC1 phosphorylates p70 S6 kinase, including S6K1 and S6K2. This promotes phosphorylation of ribosomal protein S6 and enhances ribosome biogenesis. It also increases the translational efficiency of mRNAs containing 5′-terminal oligopyrimidine (5′-TOP) motifs. These mRNAs encode ribosomal proteins and translation elongation factors. This pathway is essential for sustaining the high translational activity required for type 1 macrophages to secrete large amounts of pro-inflammatory cytokines, including TNF, IL-1β and IL-6 (64, 65).

#### 3.1.3 Context-dependent roles of mTOR signaling in the type 2 effector program

Although mTORC1 mainly promotes glycolysis and pro-inflammatory cytokine production in the type 1 effector program, its role in the type 2 tissue-remodeling effector program is more complex and context-dependent. IL-4-activated STAT6 signaling can partially maintain basal mTORC1 activity to support cell proliferation and translation of arginase 1, a core metabolic enzyme of the type 2 program. However, unlike the sustained and high-level activation of mTORC1 in the type 1 program, mTORC1 activity in the type 2 program is maintained at an intermediate level. It is also partly restrained by AMPK. This reflects the reliance of the type 2 program on fatty acid oxidation and oxidative phosphorylation for efficient ATP production. These pathways maintain a low AMP/ATP ratio, although basal AMPK activity is still preserved. This balance allows macrophages to sustain the anabolic processes required for tissue remodeling, including polyamine synthesis. It also prevents excessive glycolysis-driven skewing towards a type 1 state. In addition, IL-4 activates AKT through the mTORC2–AKT axis and activates STAT6 through the JAK–STAT pathway. The coordinated action of these pathways is required for full establishment of the type 2 effector program. Sustained AKT activation maintains basal mTORC1 activity and supports cell survival. STAT6 directly drives the transcription of type 2 signature genes, including Arg1, Mrc1/CD206 and Retnla/FIZZ1 (66, 67).

### 3.2 HIF-1α–mediated metabolic reprogramming in macrophages and the role of TLR signaling

HIF-1α is a key transcription factor governing metabolic adaptation in macrophages, and it can be activated through diverse mechanisms, including hypoxia, the metabolic intermediate succinate, ROS, and stimulation by multiple receptor-mediated signals (68). Upon activation, HIF-1α upregulates a suite of glycolysis-related genes, such as the glucose transporter GLUT1 and key glycolytic enzymes HK2, PFKFB3, PKM2, and LDH, thereby shifting macrophage metabolism from a reliance on oxidative phosphorylation toward high-rate glycolysis (69, 70). This process is often accompanied by ROS accumulation and remodeling of the tricarboxylic acid cycle. Notably, succinate and ROS are not merely metabolic by-products, they can also stabilize HIF-1α protein itself. This strengthens HIF-1α-mediated transcriptional control of type 1 effector molecules and drives activation of the NLRP3 inflammasome. Consequently, IL-1β maturation and secretion are promoted (71, 72). Together with the coordinated action of transcription factors such as NF-κB, AP-1 and interferon regulatory factors (IRFs), these metabolic and signaling events promote the establishment of the type 1 immune effector program (73).

Among the various mechanisms that activate HIF-1α, TLR-mediated signal transduction also plays a pivotal role. As pattern-recognition receptors, TLRs sense exogenous pathogen-associated molecular patterns (PAMPs) and endogenous danger signals, and they induce sustained activation of HIF-1α via downstream signaling cascades. For example, upon recognizing LPS, TLR4 augments the expression and activity of HIF-1α through MyD88- and TRIF-dependent pathways (74, 75). Thus, although TLR signaling is not the sole regulator of HIF-1α, it forges a critical coupling between metabolic reprogramming and inflammatory responses, providing a sustained stimulus that shapes macrophage function (76).

Importantly, TLR signaling also has a major role in regulating macrophages associated with the type 2 immune effector program. For example, activation of TLR2 and TLR4 can enhance the expression of type 2 effector-program markers, including Arg1 (71, 72). Thus, targeting TLR signaling may not only suppress the type 1 effector program but also promote the development of the type 2 effector program. Studies have shown that inhibition of HIF-1α-dependent TLR4 signaling reduces activation of the type 1 effector program and promotes establishment of the type 2 tissue-remodeling effector program. This provides a rationale for therapeutic strategies in chronic inflammation and impaired wound healing.

### 3.3 Regulatory role of the STAT signaling pathway in macrophage metabolic reprogramming

STAT proteins are a family of transcription factors that play a critical role in cytokine signaling and macrophage plasticity. The balance between STAT1 and STAT6 activation is a key determinant of macrophage metabolic reprogramming and effector-program direction.

Activation of STAT1 markedly enhances glycolytic metabolism by inducing IRF1 expression. IRF1 synergizes with HIF-1α to upregulate the transcription of GLUT1 and multiple key glycolytic enzymes, such as HK2, PFKFB3, PKM2, and LDH, thereby accelerating activation of the glycolytic pathway (77, 78). In addition, STAT1 strengthens glycolytic flux by promoting arginine consumption to generate nitric oxide (NO), inhibiting the activity of mitochondrial electron transport chain complex IV, inducing iNOS expression, and activating the GABA shunt metabolism (79, 80). Collectively, these processes reconfigure the TCA cycle and promote succinate accumulation. As a metabolic intermediate, succinate can stabilize HIF-1α. This creates a positive feedback loop that further amplifies glycolysis and type 1 effector signal (81). Consistent with this, heightened STAT1 activity is often accompanied by impaired OXPHOS, through mechanisms involving downregulation of genes related to mitochondrial biogenesis and exacerbated mitochondrial fragmentation (82, 83). Thus, the features of STAT1-dependent metabolic reprogramming include enhanced glycolysis, succinate accumulation, increased ROS, and constrained OXPHOS and FAO (84). This metabolic state provides macrophages with rapid energy and precursor molecules such as NO and ROS, thereby augmenting phagocytic and cytotoxic capacities (85). At the same time, glycolytic enzymes can localize around the phagosomal membrane. Through substrate-level phosphorylation, they directly provide local ATP and NADPH for phagosome maturation and NOX2-dependent oxidative burst. This creates direct spatial coupling between metabolic flux and microbicidal function (86). However, in abnormal immune microenvironments, sustained activation of the STAT1-driven type 1 effector program can promote persistent chronic inflammation. This leads to tissue damage and delayed repair (87).

By contrast, STAT6-driven metabolic reprogramming is mainly characterized by enhanced OXPHOS and FAO. This provides the metabolic basis for establishing the type 2 tissue-remodeling effector program in macrophages (88). STAT6 activation induces expression of peroxisome proliferator-activated receptor γ (PPARγ) and its coactivator PGC-1β, thereby upregulating FAO-related genes and promoting mitochondrial biogenesis and functional enhancement (89, 90). Increased FAO flux supplies ample acetyl-CoA substrate for OXPHOS, driving efficient ATP synthesis (91). Concurrently, STAT6 robustly induces ARG1 expression, facilitating the conversion of arginine to ornithine and polyamines (92). Ornithine is decarboxylated by ornithine decarboxylase 1 (ODC1) to generate putrescine, which is further converted into spermine and spermidine. These polyamines are not only essential building blocks for cell proliferation. They also support fibroblast collagen synthesis and thereby directly link macrophage ARG1 activity to ECM construction (46, 93). This metabolic branch differs from the iNOS-dominated arginine pathway associated with the type 1 effector program, which produces NO and citrulline. Instead, its products support cell proliferation, collagen deposition and tissue repair (94). Because macrophages associated with the type 2 tissue-remodeling effector program rely on OXPHOS, they show less electron transport chain leakage and do not substantially accumulate succinate. Consequently, their ROS levels are markedly lower than those of macrophages associated with the type 1 effector program (95, 96). Overall, STAT6-dependent metabolic reprogramming is characterized by high FAO/OXPHOS activity, high ARG1 expression, reduced glycolytic dependence and limited ROS production. This metabolic pattern provides a sustained and stable energy supply that supports tissue remodeling and extracellular matrix construction driven by the type 2 immune effector program (97, 98). It should be emphasized that type 2 immunity is itself a distinct inflammatory program. Its capacity to promote tissue repair under specific conditions reflects its effector-molecule profile, rather than an inherently anti-inflammatory nature. Under chronic injury conditions, persistent and excessive activation of the STAT6-dependent type 2 program can drive pathological fibrosis, as observed in liver fibrosis and idiopathic pulmonary fibrosis (99).

Notably, STAT1 and STAT6 exert antagonistic effects. STAT1 activation can inhibit STAT6 phosphorylation through iNOS-mediated nitric oxide production, thereby limiting its capacity to drive the type 2 effector program (100, 101). At the same time, STAT1-induced succinate accumulation not only reinforces the type 1 effector program by stabilizing HIF-1α but also further weakens STAT6 signaling (67, 102). Conversely, STAT6 activation can partially counteract STAT1-driven effects and promote the type 2 effector program. The dynamic balance between STAT1 and STAT6 can therefore be viewed as a key molecular node that controls the transition between type 1 and type 2 effector programs and their associated metabolic reprogramming (103). In multiple inflammatory disease models, inhibiting STAT1 activity or enhancing STAT6 signaling has demonstrated significant therapeutic potential, suggesting that targeting these pathways and their downstream metabolic enzymes may represent an important direction for immunometabolism intervention (98, 104).

### 3.4 Transcriptional regulators of macrophage metabolic reprogramming: nuclear receptors

Nuclear receptors are ligand-dependent transcription factors with important roles in macrophage effector-program control and functional regulation. Among them, PPARγ and liver X receptors (LXRs) are key regulators of macrophage effector programs (105, 106). PPARγ is a central regulator of the type 2 tissue-remodeling effector program. It promotes the expression of genes involved in immune regulation and tissue repair. PPARγ activation typically depends on endogenous ligands, including polyunsaturated fatty acids and eicosanoid derivatives, or exogenous agonists such as thiazolidinediones (102, 107). Once activated, PPARγ forms a heterodimer with retinoid X receptor (RXR). This complex binds PPAR response elements in target-gene promoters and drives the transcription of type 2 effector-program genes, including ARG1, CD206 and IL10 (108, 109). PPARγ can also suppress the activity of type 1 effector-program transcription factors, including NF-κB and AP-1. It does so through direct protein–protein interactions, competition for co-activators and induction of downstream immunoregulatory genes (110, 111). Consequently, PPARγ agonists have attracted considerable attention for their potential applications in the treatment of inflammatory diseases and in tissue repair.

Similar to PPARγ, LXRs are also critical nodes in the regulation of macrophage metabolism and inflammation (112). LXRs play central roles in cholesterol metabolism and the maintenance of lipid homeostasis (113). Upon activation by ligands such as oxysterols, LXRs promote the transcription of cholesterol-efflux genes, including ABCA1 and ABCG1. This reduces intracellular cholesterol burden, restrains excessive activation of the NLRP3 inflammasome, and enhances the immunoregulatory capacity of macrophages (106, 114). This process underscores the indispensable role of LXRs in maintaining macrophage metabolic homeostasis and preventing excessive inflammatory responses.

Moreover, the aryl hydrocarbon receptor (Ahr), another ligand-dependent transcription factor, has also been shown to participate in macrophage metabolic reprogramming (115). Although the classical function of Ahr is to mediate responses to exogenous environmental pollutants such as dioxins, recent studies indicate that it can also sense endogenous metabolites, for example kynurenine from the tryptophan metabolic pathway (116, 117). Ahr activation typically promotes an immunoregulatory response. Its downstream effects include induction of genes such as Il10 and Ido1 and suppression of type 1 effector molecules. This establishes a key link between metabolic and immune signaling (118, 119). This mechanism suggests that Ahr may serve as an important bridge in the regulation of chronic inflammation and in tissue repair.

In summary, macrophage metabolic reprogramming involves transcriptional regulatory networks mediated by multiple nuclear receptors. In addition to PPARγ, receptors such as LXRs and Ahr can sense and integrate diverse metabolic signals. They thereby coordinate the balance between inflammatory responses and metabolic state. This balance determines the direction of macrophage effector programs and their functional outcomes (93, 120).

### 3.5 Influence of metabolic products on macrophage function in the wound-healing microenvironment

As noted above, hyperglycaemia in the wound microenvironment not only alters macrophage energy metabolism but also directly induces metabolic reprogramming. It drives quiescent macrophages towards a glycolysis-dominant phenotype that is closely associated with the type 1 immune effector program (121, 122). This process primarily depends on activation of hypoxia-inducible factor HIF-1α, which upregulates the expression of glycolysis-related enzymes such as hexokinase and pyruvate kinase. Increased glycolytic flux promotes the secretion of type 1 effector molecules, including TNF and IL-1β. It also impairs oxidative phosphorylation, which provides the metabolic basis for the type 2 tissue remodeling effector program (123, 124).

ROS are not metabolites in the traditional sense but rather important by-products and signaling molecules generated during cellular metabolism. Increased ROS levels induce oxidative stress and sustain the activation of redox-sensitive transcription factors, including NF-κB and AP-1. This promotes the expression of type 1 effector molecules such as TNF-α, IL-1β and IL-6, thereby maintaining chronic inflammation within the wound (125, 126). In addition, ROS can directly impair macrophage function and survival through lipid peroxidation, protein carbonylation, and DNA damage. Meanwhile, ROS-mediated oxidative protein modifications can promote the formation of new advanced glycation end products (AGEs), amplifying the activity of the AGE-RAGE pathway and further exacerbating the inflammatory response (127, 128).

AGEs can bind to RAGE on the macrophage surface and activate multiple signaling pathways, including NF-κB and MAPK pathways. This promotes the type 1 effector program (129, 130). The AGE–RAGE axis not only enhances the transcriptional activity of type 1 effector molecules but also increases ROS production. This creates a positive inflammatory feedback loop (131). Moreover, AGEs can regulate macrophage function through epigenetic modifications; for example, they increase histone H3 acetylation at the promoters of pro-inflammatory genes (such as TNF-α and IL-1β), thereby promoting transcriptional activity (132). At the same time, AGEs downregulate the expression of phagocytic receptors MerTK and CD36, hindering the clearance of dead cells and debris and consequently prolonging local inflammation and delaying wound healing (133, 134).

In addition to ROS and AGEs, several metabolites in the wound microenvironment can markedly influence macrophage effector programs and function. First, lactate accumulates under hyperglycaemic and hypoxic conditions and can act as a signaling molecule that promotes the type 2 tissue-remodeling effector program. Lactate activates the HIF-1α and ERK pathways to upregulate angiogenic factors (e.g., VEGF), thereby facilitating resolution of inflammation and neovascularization (135, 136). Second, arachidonic acid and its derivatives, including prostaglandins and leukotrienes, regulate macrophage effector programs through NF-κB and PPARγ pathways (137, 138). Prostaglandin E2 (PGE2) is generally associated with enhanced inflammatory responses. By contrast, specialized pro-resolving lipid mediators, including resolvins, protectins and maresins, help restore the function of the type 2 tissue remodeling effector program (139, 140). Finally, adenosine, a purine metabolite, suppresses the release of type 1 effector molecules through the A2A receptor and promotes IL-10 expression. It therefore has a key immunoregulatory role during inflammation resolution (141, 142).

In sum, regulation of macrophage function during wound healing is not driven by any single factor but rather results from the concerted actions of multiple metabolites—including ROS, AGEs, lactate, lipid metabolites, and adenosine (143, 144).Through multilayered mechanisms, metabolic reprogramming, signaling pathway modulation, and epigenetic modifications, these factors shape the dynamic transitions of macrophages across the inflammatory, reparative, and regenerative phases, thereby exerting a decisive influence on the healing outcomes of chronic wounds.

## 4 Targeted therapeutic strategies for chronic wound healing and tissue repair

**Figure 3:**
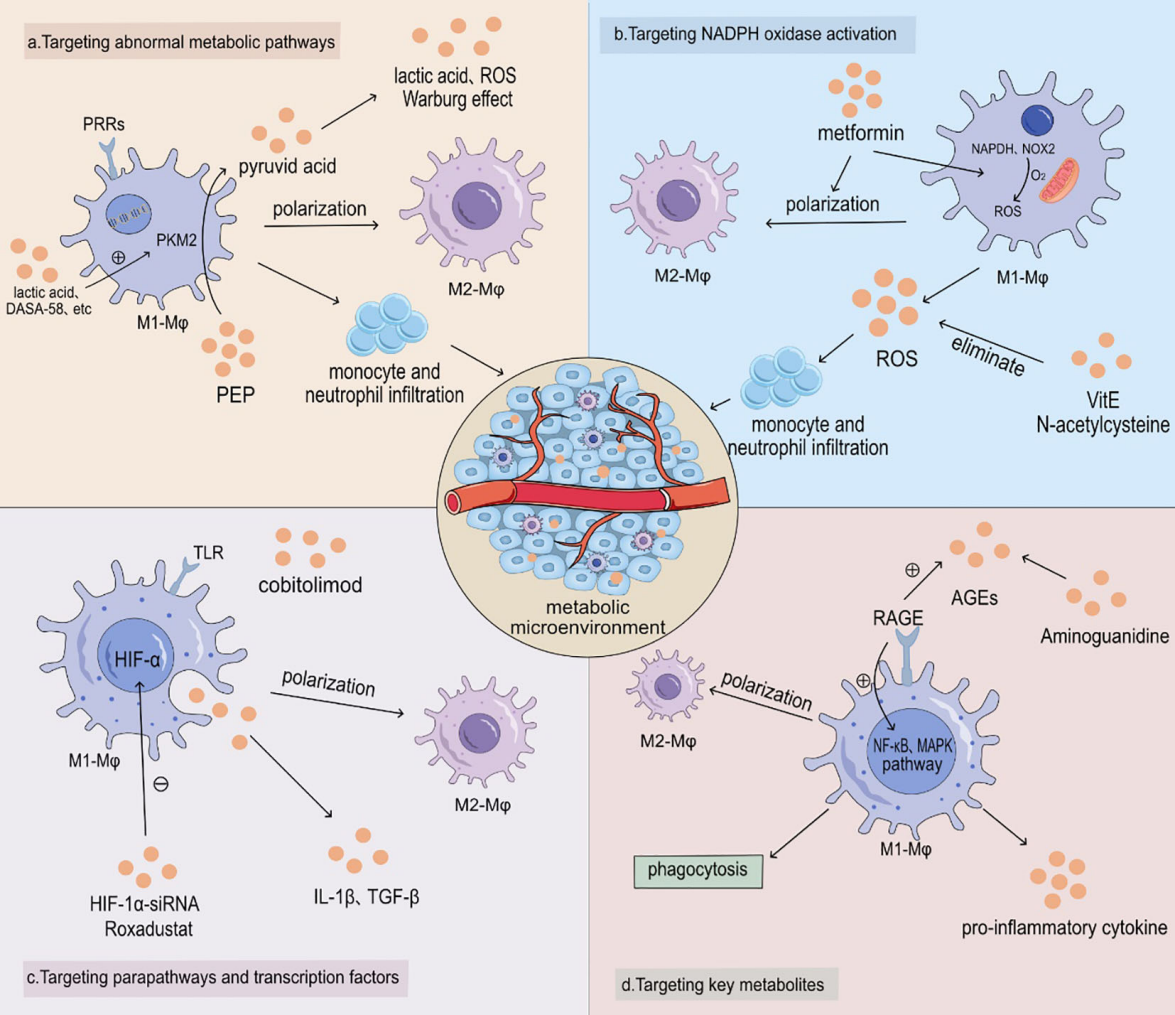
Proposed strategies for targeting the metabolic microenvironment to regulate macrophage polarization. **(a)** Targeting abnormal metabolic pathways. Modulation of lactate-, pyruvate-, phosphoenolpyruvate-, and PKM2-associated glycolytic/Warburg metabolic pathways suppresses pro-inflammatory M1 macrophage activation and promotes M1-to-M2 macrophage repolarization, thereby reducing ROS production and monocyte/neutrophil infiltration. **(b)** Targeting NADPH oxidase activation. Inhibition of NADPH oxidase/NOX2-mediated ROS generation by metformin, or ROS scavenging by antioxidants such as vitamin E and N-acetylcysteine, attenuates oxidative-stress-driven M1 activation and induces M1-to-M2 macrophage polarization. **(c)** Targeting parainflammatory pathways and transcription factors. Regulation of TLR-, HIF-1α-, and inflammation-associated signaling pathways reduces IL-1β, TGF-β, and related inflammatory/fibrotic mediators, thereby suppressing the M1 phenotype and promoting polarization toward an M2 reparative phenotype. **(d)** Targeting key metabolites. Reduction of advanced glycation end-products by aminoguanidine inhibits the AGEs–RAGE axis and downstream NF-κB/MAPK signaling, decreases M1-associated pro-inflammatory cytokine release, and promotes M1-to-M2 macrophage repolarization with enhanced phagocytic clearance. TLR, Toll-like receptor; ROS, reactive oxygen species; RAGE, receptor for advanced glycation end-products; NF-κB, nuclear factor kappa B; PKM2, pyruvate kinase M2; Warburg effect, metabolic reprogramming effect; DASA-58, PKM2 activator; TEPP-46, PKM2 activator; N-acetylcysteine, antioxidant; HIF-1α, hypoxia-inducible factor 1-alpha; IL-1β, interleukin-1 beta; TGF-β, transforming growth factor-beta; RV568, SRC family kinase inhibitor; CC-99677, MAPK inhibitor; AGEs, advanced glycation end-products; FSTL1, follistatin-like protein 1.

Chronic wounds are characterized by persistent inflammation and impaired healing, posing a significant challenge in clinical practice. Given the critical role of macrophages in maintaining immune homeostasis, directly targeting the clearance or inhibition of large numbers of macrophages may not be a safe therapeutic strategy (145, 146). However, targeting specific cellular metabolic pathways or key metabolic molecules to regulate macrophage effector-program transitions offers a new approach for treating chronic injury and promoting normal wound healing (147, 148). By focusing on the modulation of macrophage plasticity to reduce tissue damage and chronic fibrosis, this section discusses potential therapeutic strategies involving macrophage effector programs and their metabolic regulation (**Figure 3**).

### 4.1 Targeting abnormally activated glycolytic pathways to modulate M1 macrophage activation

As previously mentioned, the initiation of the inflammatory response is triggered by the recognition of DAMPs and PAMPs by PRRs on the surface of macrophages and other immune cells. This recognition rapidly drives macrophage metabolic reprogramming and shifts resting macrophages towards the type 1 microbicidal effector program. At the early stage, the local wound microenvironment can enrich factors that sustain the type 1 effector program. This promotes chronic inflammation and impairs wound healing. Elevated glucose is one such factor and has been shown to directly influence macrophage effector programs (149, 150). *In vitro* studies have shown that exposure to high glucose increases the expression of type 1 effector molecules, including TNF-α and IL-1β, in macrophages. It also suppresses the expression of IL-10 and IL-17 (151, 152). However, IL-17 should not be interpreted as an anti-inflammatory marker. IL-17 is a core pro-inflammatory cytokine of type 3 immunity. It drives neutrophil chemotaxis through the induction of G-CSF and CXCL8/IL-8 and promotes antimicrobial peptide production. Changes in IL-17 expression under high-glucose conditions should therefore be reinterpreted within the type 3 immunity framework, rather than being classified simply as changes in an anti-inflammatory marker.

The role of abnormal glycolytic pathways in tissue repair progression is significant, particularly the activity of pyruvate kinase M2 (PKM2) and its nuclear translocation, which plays a central role in disease processes. PKM2 is a key molecule in metabolic reprogramming in macrophages associated with the type 1 effector program. It has a central role in post-translational modification and glycolytic control. Recent studies have shown that lactate activates PKM2, suppresses the Warburg effect, and promotes the transition of macrophages from the type 1 microbicidal effector program to the type 2 tissue-remodeling effector program (153). Lactylation mainly occurs at the K62 site of PKM2. Lactate increases PKM2 lactylation at this site, thereby preventing the transition from tetrameric to dimeric PKM2 and enhancing pyruvate kinase activity (153). This newly defined post-translational modification of PKM2 highlights its potential value as a therapeutic target for regulating inflammatory metabolic reprogramming in macrophages associated with the type 1 effector program.

Follistatin-like protein 1 (FSTL1) is widely recognized as a secretory glycoprotein that is significantly expressed in macrophages of fibrotic livers in both humans and mice. FSTL1 directly binds to PKM2 through its FK domain, promoting the phosphorylation and nuclear translocation of PKM2, while reducing its ubiquitination. This enhances PKM2-dependent glycolysis and increases activation of the type 1 effector program. Animal studies have shown that myeloid-specific FSTL1 deletion effectively delays the progression of liver fibrosis (154). Macrophages from FSTL1^M-KO mice show suppressed type 1 effector programming and reduced activation of the NF-κB pathway both *in vivo* and *in vitro*. In mice with myeloid-specific FSTL1 deletion, infiltration of monocytes, macrophages and neutrophils is reduced. Expression of type 1 effector molecules is also decreased. These findings indicate a lower inflammatory burden during liver fibrosis progression (154). Other PKM2 activators, including DASA-58 and TEPP-46, attenuate LPS-induced activation of macrophages associated with the type 1 effector program and promote features of the type 2 tissue-remodeling effector program (155, 156). These findings suggest potential therapeutic efficacy in inflammatory diseases and other forms of endogenous injury.

### 4.2 Targeting fibrosis-associated macrophage subpopulations and related targets

Reprogramming fibrosis-associated “scar-associated macrophages” (common phenotypes: SPP1^+^/CD9^+^/TREM2^+^) from a “high-glycolysis, pro-fibrotic” metabolic state to a “fatty acid oxidation/mitochondrial oxidative, pro-resolution/clearance” state, while simultaneously restoring their capacity for efferocytosis and extracellular matrix degradation, can promote scar absorption and fibrosis regression (**Table 1**).

**Table 1:** Fibrosis-associated macrophage subsets and targets for fibrosis reversal.

Macrophage subsets	Core markers	Origin	Major expression tissues	Metabolic features	Functional state	Therapeutic targets	References
TREM2^+^ CD9^+^SAMs	TREM2, CD9	CD14^+^ monocytes in blood circulation	Enriched in fibrotic lesions of liver, lung, kidney, and skin	Glycolysis-dependent pro-fibrotic program; upregulation of lipid metabolism, cholesterol/lipoprotein handling, lysosomal and phagocytic pathways (APOE, LPL, FABP5); frequently accompanied by enhanced oxidative phosphorylation activity	Tissue-dependent functional state; strengthens fibroblast activation and ECM deposition; promotes type I collagen deposition; remodels basement membrane and facilitates scar expansion	TREM2–SYK–PI3K–AKT–mTOR axis; cholesterol esterification enzyme SOAT1; LXR–ABCA1/ABCG1 pathway	(159, 163, 183, 184)
SPP1^+^GPNMB^+^FABP5^+^ macrophages	SPP1 , GPNMB, FABP5, CD63	Infiltrating macrophages derived from peripheral monocytes (non-homeostatic, distinct from resident KCs, AMs)	Enriched at fibrotic scar edges and along the outer side of fibrous bundles, in close proximity to activated fibroblasts/myofibroblasts and collagen deposition zones	Active oxidative phosphorylation; lipid handling and lysosomal pathways upregulated, with high expression of LPL, APOE, FABP5 and lysosome/phagocytosis-related genes	A conserved feature across multi-organ fibrosis; pro-fibrotic paracrine axis via secretion of SPP1 acting on fibroblasts through CD44 and integrins αvβ3/β5, promoting αSMA/FAP upregulation and collagen I deposition	lockade of the SPP1/receptor axis; GPNMB–lysosomal stress pathway; PPARγ–FABP5 axis, CXCL4–platelet axis	(166, 168, 180, 181)
TREM2^+^ CD9^+^LAMs	TREM2, CD9, SPP1, GPNMB, ABCA1, ABCG1	Monocytes in blood circulation	Enriched in fibrotic lesions such as skin fibrosis and liver cirrhosis	Strongly enhanced lipid uptake and processing, with upregulation of LPL, LIPA, FABP5, and APOE; strengthened cholesterol efflux and transport via active ABCA1/ABCG1 pathway; robust lysosome–lipid droplet crosstalk and tissue clearance programs, with elevated lysosomal activity of CTSB/CTSL, CST7, and GPNMB, supporting a “scavenger/de-lipidation” profile	Fibrotic activity is stage- and tissue-dependent—during progression, they may adopt a pro-fibrotic SPP1^+^ scar-associated state, whereas in regression/repair, the TREM2-dependent LAM program facilitates fibrosis reversal	PPAR axis; LXR–ABCA1, ABCG1 pathway; TREM2, SPP1	(160, 174, 177, 178)
CCR2-dependent monocyte-derived SPP1^+^ macrophages	CD9, TREM2, SPP1, GPNMB, FABP5	Recruitment of peripheral monocytes via CCR2	Enriched in fibrotic lesions such as skin fibrosis and liver cirrhosis	Upregulated lipid metabolic programs with active FABP5 and APOE lipid transport and processing genes; under hypoxic inflammatory conditions; HIF-1α–driven glycolysis is enhanced	Pro-fibrotic remodeling; secrete SPP1, MMPs and matrix proteins; engage strongly with fibroblasts to drive their activation and ECM deposition; overall associated with poor prognosis	FABP5–PPARγ axis; LXR pathway; glycolysis, HIF-1α–GLUT1 module	(158, 183, 233, 234)

Scar-associated macrophages (SAMs), lipid-associated macrophages (LAMs).

#### 4.2.1 Inhibition of macrophage glycolysis: the HIF-1α–PFKFB3 axis

High glycolytic activity supports the pro-inflammatory and pro-fibrotic phenotype driven by the type 1 effector program. In the murine renal fibrosis (UUO) model, knockdown/inhibition of macrophage PFKFB3 significantly attenuates fibrosis (157, 158). Recent studies observed a positive correlation between the expression of 6-phosphofructo-2-kinase/fructose-2,6-biphosphatase 3 (PFKFB3) and the severity of renal fibrosis (159, 160). Specific deletion of PFKFB3 markedly reduced renal lactate levels in IRI mouse models, thereby alleviating inflammation and fibrosis (161, 162). Mechanistically, lactate derived from PFKFB3-mediated tubular glycolytic reprogramming significantly enhances histone lactation, particularly at the promoters of NF-κB signaling genes, activating their transcription and promoting inflammatory responses. Thus, targeting the PFKFB3-mediated NF-κB signaling pathway may represent a novel strategy for reversing tissue fibrosis. Through PFKFB3 inhibition, moderate suppression of glycolytic flux, and reduction of lactate-driven NF-κB/IL-1β activation, fibrosis can be significantly mitigated. However, potential systemic side effects necessitate targeted delivery (159, 163). Notably, PFKFB3 locally drives glycolytic ATP production at the leading edge of migrating macrophages. This directly fuels actin polymerization and pseudopod extension. This spatial coupling suggests that PFKFB3 inhibition may have an additional role in regulating macrophage migration and infiltration (164, 165).

#### 4.2.2 Enhancement of OXPHOS/FAO: AMPK–PPAR–mitochondrial reprogramming

Myeloid AMPK signaling limits fibrosis (166). Metformin-mediated AMPK activation suppresses TGF-β/Smad signaling and fibroblast activation in multiple models of fibrosis. It also shifts macrophages towards an OXPHOS-dominant type 2 tissue-remodeling and pro-resolving effector program (167). Metformin-activated AMPK can downregulate FOXM1 and attenuate bleomycin-induced pulmonary fibrosis (PF) in mice (168). *In vitro*, AMPK activation weakened PDGF-induced fibroblast proliferation, accompanied by FOXM1 downregulation (169, 170). Conversely, AMPK inhibition enhanced PDGF-induced fibroblast proliferation and simultaneously activated FOXM1 (171). These findings suggest that AMPK can improve fibroblast proliferative progression during PF by suppressing FOXM1 expression, offering new insights for therapeutic strategies in fibrotic diseases.

#### 4.2.3 Activation of “pro-resolving lipid mediators”: the SPM pathway

Specialized pro-resolving mediators (SPMs) can reprogram macrophage metabolism and function, enhancing phagocytosis while suppressing inflammatory responses, and in animal studies this is accompanied by a reduction in fibrosis burden (172). SPMs are endogenous small molecules, primarily derived from dietary omega-3 polyunsaturated fatty acids and produced by structural cells as well as active and innate immune cells (173, 174). These specialized pro-resolving mediators have been shown not only to limit acute inflammation but also to promote resolution after infection or injury and restore homeostasis (175). Increasing evidence indicates that chronic injury and fibrosis are characterized by insufficient SPMs, while SPMs hold tremendous potential as novel preventive and therapeutic agents for chronic inflammation and fibrotic diseases (176, 177). Thus, strategies may include boosting omega-3 precursor supply or employing stable SPM analogs as innovative therapies for chronic injury and fibrotic disorders. It should be noted that the full molecular mechanisms of active resolution remain incompletely defined. Resolution is not merely the cessation of pro-inflammatory mediator production. Rather, it is an active process that requires metabolic support, such as fatty acid oxidation, which provides substrates for resolvin biosynthesis.

#### 4.2.4 Targeting “scar-associated” macrophages: osteopontin (SPP1^+^) macrophages

SPP1^+^ macrophages are enriched in multi-organ fibrosis, and inhibition of SPP1 or its differentiation pathways can alleviate fibrotic burden across various models (178). TREM2^+^ macrophages play context-dependent roles across organs and disease stages, requiring careful interpretation (179, 180). In progressive metabolic dysfunction-associated steatohepatitis (MASH), resident Kupffer cells are lost and replaced by monocyte-derived macrophages (MoKCs, LAMs, etc.); in regression phases, no novel subpopulations emerge, but macrophage composition is remodeled: MoKCs decrease, while LAMs become dominant and sustain Trem2 expression (179).

TREM2 is critical for LAM formation and crown-like structure establishment; its absence blocks fibrosis and inflammation resolution, thereby hindering the reversal of steatosis and hematopoietic stem cell inactivation. TREM2^+^ macrophages exert protective effects by enhancing phagocytosis, lipid handling, and collagen degradation, making them central effectors in limiting MASH fibrosis progression and promoting regression (181). Thus, the absence of TREM2 impedes both LAM emergence and hepatic crown-like structure formation. Beyond their role in restricting fibrosis progression, TREM2^+^ macrophages are essential for efficient inflammation and fibrosis regression (182). They are superior collagen degraders, and their deficiency also prevents steatosis regression and hematopoietic stem cell inactivation during resolution, underscoring their importance in metabolic coordination with other hepatic cell types (183). Through multifactorial mechanisms, including improved phagocytosis, lipid processing, and collagen degradation, TREM2 endows macrophages with these protective functions (184).

#### 4.2.5 Promoting ECM degradation: macrophage “collagen brushing”

Fibrosis is essentially an aberrant continuation of the tissue repair process, characterized by excessive extracellular matrix (ECM) deposition, primarily collagen, ultimately leading to tissue dysfunction and organ failure. During the resolution phase, scar-associated macrophages can secrete collagenases/proteases such as MMP-13 and MMP-9, and, in synergy with inhibitors of matrix cross-linking enzymes (e.g., LOXL2 inhibitors), accelerate collagen degradation and scar absorption (185).

Inflammation, oxidative stress, cytokines, and matrix remodeling play critical roles in the initiation and progression of fibrosis, with matrix metalloproteinases (MMPs) serving as the central drivers of ECM degradation and regarded as important therapeutic targets for anti-fibrosis interventions. In contrast, tissue inhibitors of metalloproteinases (TIMPs) act as endogenous counter-regulators to maintain balance (186). Previous studies have shown that MMP-9 is particularly pivotal in fibrosis regulation; however, its effects are bidirectional and may vary depending on disease type or stage, displaying either pro-fibrotic or anti-fibrotic outcomes (187, 188). Current MMP-9 inhibitors such as pirfenidone and nintedanib have demonstrated therapeutic efficacy in clinical applications but are accompanied by notable side effects (189, 190). Therefore, deeper mechanistic insights into MMP-9 function and its signaling pathways across different tissues and stages of fibrosis will provide novel avenues for the development of safer and more precise biologics.

### 4.3 Targeting mitochondrial dysfunction and NADPH oxidase activation

Oxidative stress is a key feature of the post-injury immune microenvironment and has an important role in regulating macrophage effector programs. Changes in the metabolic microenvironment induce mitochondrial dysfunction and activate NADPH oxidases, leading to marked increases in ROS, including superoxide and hydrogen peroxide, at the injury site (191). Sustained ROS production activates type 1 effector-program signaling pathways in macrophages, including NF-κB. Moreover, ROS impairs the transition of macrophages towards the type 2 tissue-remodeling effector program, a process essential for wound healing (192, 193). Therefore, regulating the levels of ROS at the injury site represents a promising area of research.

Studies have shown that antioxidants such as N-acetylcysteine and vitamin E effectively reduce oxidative stress levels and promote wound healing in animal models (194, 195). These antioxidants not only scavenge ROS but also suppress activation of type 1 effector-program signaling pathways. They thereby promote the transition of macrophages towards the type 2 tissue remodeling effector program (196, 197). Clinical trial results have also confirmed that the local application of antioxidants significantly improve wound healing outcomes (198).

In addition to antioxidants, regulating macrophage metabolism represents a potential therapeutic strategy (199). Compounds such as resveratrol and metformin have been shown to promote oxidative phosphorylation and mitochondrial biogenesis. This facilitates the transition of macrophages towards the type 2 tissue remodeling effector program and improves wound healing in diabetic animal models (200, 201). These compounds activate AMPK, promote the expression of immunoregulatory genes and suppress glycolysis (202). Clinical trials have also demonstrated that metformin significantly enhances wound healing in diabetic patients, as it not only improves wound closure rates but also effectively reduces inflammation (203, 204).

### 4.4 Targeting signaling pathways and transcription factors to regulate macrophage polarization

Targeting specific signaling pathways and transcription factors to regulate macrophage function has emerged as a potential therapeutic strategy to promote tissue repair and regeneration. Studies have shown that TLR9 signaling can induce macrophages to shift towards a type 2 tissue-remodeling and immunoregulatory effector program in several models (205, 206). For example, agonist-mediated activation of TLR9 promotes a pro-healing macrophage effector program. This enhances macrophage-mediated tissue repair in diseases such as ulcerative colitis (207). MAPK signaling also has a key role in macrophage activation and effector program control (208). Inhibition of these pathways has been shown to promote the type 2 effector program and reduce inflammation. Drugs such as RV568, an SRC family kinase inhibitor, and CC-99677 suppress MAPK pathway activity, reduce inflammatory cytokine production and promote the transition of macrophages towards the type 2 effector program (209, 210).

Moreover, the key transcription factor HIF-1α plays an important role in regulating macrophage glucose metabolism in models of diabetic nephropathy and acute kidney injury. Treatment of macrophages with HIF-1α siRNA or the prolyl hydroxylase inhibitor, roxadustat, or direct inhibition of macrophage glycolysis using 2DG significantly reduces the gene expression of inflammatory cytokines such as IL-1β and fibrosis-promoting factors like TGF-β (211). Roxadustat not only suppresses the inflammatory response in macrophages and neutrophils, protects tissue cells, and alleviates tissue damage induced by chronic inflammation, but also activates HIF-1α in cisplatin-induced acute tissue injury models (212, 213). In addition, in a model of hard-to-heal diabetic foot ulcers, ABCB5-positive dermal mesenchymal stromal cells suppress HIF-1α and GLUT1 expression and block macrophage glycolysis. This markedly inhibits activation of macrophages associated with the type 1 effector program and produces strong anti-inflammatory effects. It also promotes wound healing by inducing angiogenesis (214). Therefore, modulating the activity and degradation of HIF-1α holds significant therapeutic potential, making it a promising therapeutic target.

### 4.5 Targeting key metabolites such as glucose, ROS, and AGEs to regulating macrophages associated with the type 2 tissue remodeling effector program

AGEs are substances formed through the non-enzymatic glycation of proteins and lipids under hyperglycemic conditions. They gradually accumulate in wound tissue and hinder the healing process (215). When AGEs bind to receptors on the macrophage surface, including the receptor for advanced glycation end products (RAGE), they activate type 1 effector-program signaling pathways such as NF-κB and MAPK. This triggers inflammatory responses and promotes the production of ROS and type 1 effector molecules (216). The interaction between AGEs and RAGE not only exacerbates macrophage inflammatory activation but also impairs phagocytic function, which is essential for apoptotic cell clearance and inflammation resolution (217). Thus, AGE accumulation in wound tissue sustains chronic inflammation and markedly impairs the transition of macrophages towards the type 2 tissue-remodeling effector program.

An effective strategy is to inhibit the formation of AGEs and their accumulation at the wound site. Studies have shown that drugs such as aminoguanidine and pyridoxamine can effectively reduce AGE formation and promote wound healing in animal models (218, 219). These compounds work by capturing reactive carbonyl intermediates and preventing their binding to proteins, thereby reducing AGE formation (220). Furthermore, interventions targeting the AGE-RAGE signaling pathway, such as using RAGE antagonists or soluble RAGE (sRAGE), have shown promising results in preclinical studies (221). As a decoy receptor, sRAGE can sequester AGEs and prevent their binding to cell-surface RAGE. This suppresses type 1 effector-program signaling in macrophages (222).

### 4.6 Targeting mTOR signaling to globally regulate macrophage metabolic state

The multidimensional regulatory functions of mTOR make it a unique target for broad rewiring of macrophage metabolism. Unlike interventions that act on a single metabolic pathway, mTOR modulation can affect several metabolic layers at once. It can suppress inflammatory damage driven by the type 1 effector program while also modulating fibrosis driven by the type 2 effector program.

Rapamycin, also known as sirolimus, binds the immunophilin FKBP12 and allosterically inhibits the FRB domain of mTORC1. Its metabolic effects in macrophages occur at three levels. First, rapamycin blocks the mTORC1–4E-BP axis. This reduces HIF-1α translation and decreases glycolytic enzyme expression, thereby suppressing the pro-inflammatory activity of macrophages associated with the type 1 effector program (223). Second, rapamycin releases TFEB from mTORC1-dependent inhibitory phosphorylation. This promotes TFEB nuclear translocation and drives the transcription of lysosomal and autophagy genes. It thereby enhances macrophage clearance of apoptotic cells and debris and promotes inflammation resolution. Third, rapamycin limits proteolytic activation of SREBP1/2 and moderately reduces *de novo* lipid synthesis. This restrains excessive activation of the NLRP3 inflammasome, whose assembly depends on specific lipid microenvironments (224). Notably, rapamycin has weaker effects on mTORC2. It only indirectly disrupts mTORC2 assembly after prolonged exposure. This may partly preserve macrophage chemotactic capacity and could therefore have protective relevance in tissue repair. Second-generation mTOR kinase inhibitors, including Torin1, AZD8055 and INK128, target both mTORC1 and mTORC2 through ATP-competitive inhibition. These agents provide more complete suppression of the type 1 effector program. However, because they also inhibit the mTORC2–AKT axis, they can impair macrophage migration and survival. Their therapeutic safety window is therefore narrower (225, 226).

Although mTOR targeting has substantial potential for regulating macrophage metabolism, its clinical translation faces several key challenges. The first is cell-type specificity. mTOR has essential functions in almost all cell types. Systemic mTOR inhibition may therefore cause broad immunosuppression and metabolic adverse effects. The second is spatiotemporal control of the therapeutic window. mTORC1 has distinct roles at different stages of wound healing. During the inflammatory phase, moderate mTORC1 inhibition helps restrain excessive type 1 effector programming. However, during the proliferative and repair phases, basal mTORC1 activity is required for type 2 program-driven cell proliferation, polyamine synthesis and collagen production. Combination therapy is therefore often considered. Combining mTOR targeting with modulation of other metabolic nodes may produce synergistic effects. For example, mTORC1 inhibition can reduce glycolysis, whereas PPARδ agonism can enhance fatty acid oxidation. Together, these interventions may more efficiently guide the transition from the type 1 to the type 2 effector program (227).

## 5 Conclusion and future outlook

Macrophages are highly plastic immune cells with central roles in maintaining tissue homeostasis, coordinating inflammatory responses, and driving tissue repair and regeneration (228). Their activation states should not be understood through the historical M1/M2 binary. Rather, they occupy a continuous functional spectrum. This spectrum can be described along three interconnected axes. The first is the effector axis. Macrophages dynamically shift between cytotoxic effector functions and tissue remodeling functions. The former corresponds to the type 1 immune program, whereas the latter mainly corresponds to the type 2 immune program. The second is the metabolic axis. The type 1 cytotoxic state depends on rapid glycolysis to supply energy and biosynthetic precursors. The type 2 tissue remodeling state is associated with enhanced OXPHOS, lipid metabolism and amino acid metabolism. At specific pathological stages, glycolysis and OXPHOS can be activated simultaneously, generating a mixed metabolic phenotype. The third is the contextual axis. Continuous changes in effector programs are constrained by tissue origin, the timing of inflammation and the physicochemical microenvironment. For example, glycolysis and lipid metabolism are often rewired in hypoxic tumors. In acute injury, the type 1 program dominates early, whereas macrophages gradually shift towards type 2 tissue remodeling during the later repair phase (229, 230). Type 3 immunity, driven by IL-17, has a distinct role in infected wounds and mucosal barrier repair. It promotes neutrophil chemotaxis and antimicrobial peptide production. However, its metabolic support mechanisms remain poorly defined and represent an important area for future research.

At the level of intervention, single-cell multi-omics and metabolic flux analysis in target tissues can be used to monitor key metabolites, including succinate, fumarate, lactate, cholesterol esters and oxidized lipids. These readouts can help define the inflammatory functional state of the tissue microenvironment and identify metabolic transitions. On this basis, time sequenced therapeutic strategies can be used to intervene differently during the inflammatory initiation phase and the resolution and repair phase. For example, early PI3Kγ inhibition combined with other therapies may relieve immunosuppression. This could then be followed by interventions that regulate the PPAR axis and mitochondrial quality-control programs (231, 232). Several core challenges remain. It is still unclear how specific metabolic pathways, including glycolysis, FAO, glutaminolysis, polyamine synthesis and the citrate–ACLY axis, selectively support defined effector outputs such as phagocytosis, migration, cytokine secretion and ECM construction, rather than broadly supporting a general phenotype. Most current evidence is derived from *in vitro* studies, and the route to clinical translation remains uncertain. Moreover, metabolic reprogramming is shaped by both microenvironmental and interindividual variation. The rules governing effector-program transitions across different disease contexts therefore require systematic investigation.

In summary, targeting metabolic reprogramming to regulate macrophage effector programs remains at an early stage, but its therapeutic promise is clear. The aim should not be to lock macrophages into a fixed phenotype. Instead, macrophages should be viewed as a metabolic and functional continuum that changes across time and microenvironmental contexts. This continuum may be precisely controlled through stratified monitoring, time-sequenced intervention and localized combination therapy. Future research should first establish direct causal links between metabolic flux and specific effector functions, including phagocytosis, migration, cytokine secretion and ECM construction. It should also define the metabolic support mechanisms of type 3 immunity-associated macrophages. In parallel, real-time *in vivo* metabolic flux monitoring technologies should be developed to enable dynamic tracking and precise intervention of macrophage effector programs. Finally, the efficacy and safety of metabolism targeted strategies should be systematically validated in preclinical models of fibrosis and chronic wounds.
